# Adipocyte Size, Overweight, and Insulin Resistance in Type 2 Diabetes Mellitus and the Impact of Weight Loss: A Systematic Review

**DOI:** 10.3390/nu18091382

**Published:** 2026-04-28

**Authors:** Kuat P. Oshakbayev, Altay N. Nabiyev, Aigul K. Durmanova, Gani M. Kuttymuratov, Timur S. Suleimenov, Nurzhan A. Bikhanov, Alisher S. Idrissov, Guldana Zh. Bazheneyeva, Kenzhekyz Manekenova, Ainur R. Akilzhanova, Bibazhar A. Dukenbayeva

**Affiliations:** 1 Clinical Academic Department for Internal Medicine, University Medical Center, Syganak Str., 46, Astana 010000, Kazakhstan; a.nabiyev@umc.org.kz; 2 Center for Endocrinology, University Medical Center, Syganak Str., 46, Astana 010000, Kazakhstan; aigul.durmanova@umc.org.kz; 3 Center for Surgery, University Medical Center, Syganak Str., 46, Astana 010000, Kazakhstant.suleimenov@umc.org.kz (T.S.S.); n.bihanov@umc.org.kz (N.A.B.); 4 Department of Family Medicine, Astana Medical University, Beibitshilik Str., 49a, Astana 010000, Kazakhstan; idrisov.a@amu.kz; 5 Research Department, University Medical Center, Turan Str., 38, Astana 010000, Kazakhstan; g.bazheneeva@umc.org.kz; 6 Department of Pathology, Astana Medical University, Beibitshilik Str., 49a, Astana 010000, Kazakhstan; manekenova.k@amu.kz; 7 Laboratory of Genomic and Personalized Medicine, Center for Life Sciences, National Laboratory Astana, Nazarbayev University, 53, Kabanbay Batyr Ave, Astana 010000, Kazakhstan; akilzhanova@nu.edu.kz

**Keywords:** type 2 diabetes mellitus, Insulin resistance, endogenous hyperinsulinism, HbA1c, obesity paradox, overweight (growing), overweight (maximum), weight loss

## Abstract

**Background:** The impact of overweight and adipocyte size on the development of type 2 diabetes mellitus (T2DM) remains unclear. **Aim:** We studied (1) the relationship between the state of adipocytes and/or overweight/obesity, the development of T2DM and its clinical and laboratory features; and (2) weight loss effect on glycemic level, endogenous hyperinsulinism (HI), insulin resistance (IR), and T2DM. **Methods:** We designed a systematic review by searching Web of Science, EBSCO, Scopus/ Science-Direct, Google Scholar, PubMed, Cochrane, and Wolter Kluwer for articles published in 26 years (2000–2026). The study was based on a systematic review of 3853 articles published worldwide. **Results:** In total, 142 full-text articles were assessed for eligibility. As overweight increases, the size of adipose tissue, adipocytes, and cell radius increase. The increase in cell size overloads intracellular transport and internal organs. The development of IR is a conformational change in cellular receptors caused by an excessive increase in cell size. The increase in cell size with overweight gradually leads to hyperglycemia and HI with the development of IR and T2DM. Any targeted intentional weight loss in patients with T2DM improves metabolic and cardiovascular health, reduces blood pressure and blood sugar, and decreases HI, IR, and T2DM. **Conclusions:** IR is a protective response of cells that prevents oversaturation and overflow. Overweight is an independent risk factor for the development of HI, IR, and T2DM. Targeted weight loss leads to the cure of HI, IR and T2DM.

## 1. Introduction

Type 2 diabetes mellitus (T2DM) is a global socio-clinical public health problem affecting more than 450 million people worldwide, [US Centers for Disease Control and Prevention, national diabetes statistics report, 2024. https://www.cdc.gov/diabetes/php/data-research/index.html, accessed on 1 January 2026] with high morbidity and mortality rates in both adults and children [1,2,3,4].

Over the past twenty years, a growing body of clinical evidence has demonstrated the remission of clinical and laboratory T2DM markers following a reduction in body fat mass [5,6]. This has sparked a surge in weight-loss interventions for T2DM patients. Currently, three main evidence-based approaches dominate the field: pharmacological therapy [7,8], bariatric surgery [9,10], and low-calorie diets combined with physical activity [11]. While widely discussed in both the scientific and popular literature, each method presents distinct advantages and disadvantages [10,12].

Recent research suggests that traditional long-held views of the causes of T2DM—as a progressive, incurable chronic disease caused simply by “lifestyle” or excessive sugar intake, or as growing molecular and genetic evidence complicates the understanding of the disease’s nature—may be limiting treatment success [13]. The conventional treatment paradigm often focuses on lowering blood glucose (HbA1c) rather than addressing the disease’s underlying etiology, frequently resulting in suboptimal patient outcomes [14]. Consequently, some authors have argued that we have historically misunderstood the development of T2DM, leading to incorrect treatment strategies [8,9,15].

Many patients with T2DM fail to maintain glycated hemoglobin (HbA1c) within the American Diabetes Association’s target range [8,16], despite the increased use of glucagon-like peptide-1 receptor agonists (GLP-1RA) and sodium–glucose co-transporter-2 inhibitors (SGLT-2i) [17].

Physiologically, normal blood glucose level is maintained by insulin under constantly changing conditions in accordance with a cybernetic feedback system through dynamic alignment. However, long-term exogenous insulin therapy in diabetes eventually suppresses pancreatic beta-cell function and exacerbates insulin resistance (IR), necessitating higher doses of exogenous insulin [18,19]. Therefore, it is essential to move beyond merely increasing insulin availability (whether through administration or stimulation). Hyperinsulinism (HI) in the context of IR may not be a purely pathological state requiring immediate suppression, but rather a compensatory mechanism whose underlying causes require further investigation [20,21].

Furthermore, the simplistic view that sugar consumption is the sole driver of T2DM is not supported by data: while glucose is metabolized, only fat accumulates in the body. Today, the pathogenesis of T2DM is recognized as far more complex than simple insulin depletion or beta-cell exhaustion [6,18,22].

There is still no consensus regarding the primary cause of the metabolic disturbances in the pathogenesis of T2DM. Some authors have argued that T2DM develops as a consequence of long-term essential hypertension, which reduces peripheral blood flow and triggers the development of IR [15,23,24]. In this view, T2DM, hypertension, and endothelial dysfunction are typically the downstream effects of being overweight [25]. Conversely, other researchers have suggested that a hereditary predisposition to IR and obesity—compounded by physical inactivity and overnutrition—drives tissue IR and subsequent compensatory hyperinsulinism [23,26,27,28].

Extensive evidence also identifies central obesity as a major driver of IR, HI, and broader metabolic disorders. Adipocytes in visceral adipose tissue secrete free fatty acids directly into the hepatic portal vein, where these high concentrations suppress hepatic insulin uptake, resulting in systemic HI and relative IR [7,8]. Another theory has linked the pathogenesis of IR in obesity to alterations in adipokine levels, such as leptin, ghrelin, and adiponectin [29]. These hormones, synthesized by adipocytes in adipose tissue, correlate closely with body mass index (BMI) and regulate satiety via the brain’s subcortical nuclei [30,31]. As adipocyte hypertrophy alters these hormone levels in the blood (adipokines) [32,33,34], targeted weight reduction has been shown to normalize these endocrine profiles [35,36].

In patients with T2DM, hyperlipidemia and dyslipidemia occurring alongside adipocyte hypertrophy [33,37] gradually trigger systemic atherosclerotic changes. These changes contribute to the development of renovascular hypertension, psoriasis, non-alcoholic fatty liver/pancreas disease, and systemic inflammatory conditions [29,38,39,40].

However, several fundamental questions remain: Why does excess weight eventually progress to IR and the subsequent onset of T2DM [29,41]? Why is T2DM almost universally associated with being overweight, yet not always with obesity—which, interestingly, often plateaus or declines as the disease progresses [42]? Furthermore, how can we account for the “obesity paradox”, which suggests that increased body mass may offer protective effects and reduce mortality in certain chronic disease populations [43,44]? Finally, what are the specific pathophysiological mechanisms through which targeted weight loss leads to the normalization of blood glucose levels [45,46,47,48]? These unresolved questions regarding the pathogenesis of T2DM form the basis of this systematic review. The purpose of this study is to examine: (1) the relationship between adipocyte morphology, overweight/obesity, and the clinical and laboratory features of T2DM; and (2) the specific impact of weight loss on glycemic levels, HI, IR, and overall T2DM management.

## 2. Methods

### 2.1. Design and Registration

This systematic review was conducted in accordance with the PRISMA 2020 statement [49]. Details of the historical subject matter and clinical/research practice, including the combined inductive and deductive analyses, results, authors’ reflections, and lessons learned, are applicable.

### 2.2. Review Questions and Search Strategy

Two research questions of the systematic review were: (1) The inductive question (cause)—what is the effect of increasing adipocyte size and/or overweight/obesity on the development of IR and HI in T2DM? (2) The deductive question (effectiveness)—what is the effect of weight loss on glycemia, HI, IR, and T2DM?

We searched seven electronic databases (Web of Science Core Collection/pre-Prints/EndNoteClick/Kopernio/Medline, EBSCO/Medline-Complete, Scopus/EMBASE/Science-Direct, Google Scholar, NCBI/PubMed, Cochrane/CENTRAL, и Ovid/Wolter Kluwer). We considered HbA1c, HI, and IR as parameters of T2DM. The search results were recorded, analyzed, and the selection criteria were applied. Articles identified during the initial database review were assessed for inclusion/exclusion criteria if they represented original, peer-reviewed epidemiological and clinical studies conducted on humans or animals. There were no language restrictions. The search was repeated before the final analysis (qualitative/quantitative assessment), and eligible studies were selected for inclusion in this study. All articles considered eligible for this systematic review were required to provide data on adipocyte size, BMI, HbA1c, and development of IR, HI, and T2DM, as well as changes in these parameters after weight loss.

Primary endpoints were an increased adipocyte size; overweight/obesity, IR, and T2DM; and weight loss. Secondary endpoints were HI, HbA1c, and lipids.

### 2.3. Inclusion and Exclusion Criteria

For study inclusion, the first question of the systematic review included epidemiological, observational, cohort, cross-sectional, case–control studies, systematic reviews, and meta-analyses. The second question of the systematic review included randomized clinical trials, prospective controlled interventional studies, experimental studies, systematic reviews, and meta-analyses. The search for published studies covered the period from January 2000 to January 2026. For the primary literature retrieved, abstracts were screened to identify publications reporting our primary and secondary endpoints. If the data were not reported in the abstract, the full text was screened using the same criteria.

The search used a combination of MeSH terms and keywords, both together and individually: increased adipocyte size and number (both exposure and outcome); weight change; overweight/obesity (exposure and outcome); HbA1c (outcome); HI (outcome); IR (outcome); T2DM (outcome); dyslipidemia/hyperlipidemia (outcome); “obesity paradox”; body potential energy and capacity for weight gain (exposure and outcome); and weight loss/gain (exposure and outcome). From the included studies, we selected full-text articles demonstrating the influence of adipocyte size, body weight, and/or overweight/obesity on the development of IR, HI, and T2DM. We briefly discussed the interactions between the exposures and outcomes, as well as the impact of targeted weight loss on these outcomes.

The exclusion criteria included articles assessing glycemic parameters in inherited diseases; conference abstracts; book chapters; thesis/dissertations; case reports; editorials; and articles that did not report any of our pre-specified primary and secondary outcomes.

### 2.4. Quality Assessment of the Included Studies

**Figure 1:**
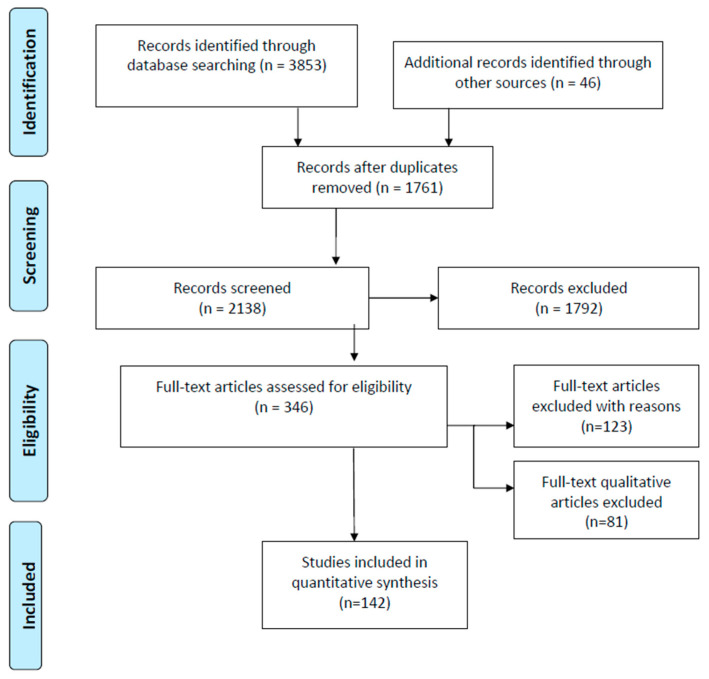
PRISMA flow diagram of the data collection process.

Three researchers (K.P.O., B.A.D., and G.M.K.) independently assessed a paper report form for each the titles and abstracts of the studies and then reached an agreement on the included studies and extracted data, according to the study inclusion/exclusion, with the other three authors (A.K.D., A.N.N., and A.S.I.). Results were exported to Endnote 20ver, and duplicates were removed. Disagreements were resolved through discussion between two authors, while a third author helped resolve. The researchers considered the validity and rigor of the study, the reliability of the results, the generalizability or applicability of the results, and how useful and relevant the results included in the study were. Three researchers (K.P.O., B.A.D., and G.M.K.) conducted the data analysis, and the other three researchers (N.A.B., T.S.S., and K.M.) reviewed, verified, and validated the results. The final number of included records was decided by all researchers. The reasons for exclusion were recorded and reported in Figure 1.

### 2.5. Risk of Bias

The included studies were assessed for risk of bias using two specific tools: the Cochrane RoB-2 for randomized control trials (RCTs) and the Cochrane ROBINS-I-V2 tool for non-RCTs. RCTs were evaluated across five domains: the randomization process, deviations from intended intervention, missing outcome data, measurement of the outcome, and selection of the reported result. Non-RCTs were assessed for bias across six domains: confounding, classification of intervention, selection of participants into the study, missing data, measurement of outcomes, and selection of the reported result. We included both RCTs and non-RCTs to comprehensively compare the effects of various weight-loss modalities—pharmacological, dietary, and surgical—on hyperglycemia, HI, IR, and T2DM. This inclusive approach was necessary because randomization often lacks clinical equipoise in surgical contexts or is logistically unfeasible [50,51]. Notably, surgical interventions often result in the most significant weight loss, making their inclusion vital for this analysis despite the inherent challenges of randomization in surgical research.

**For ethical approval**, the Ethical Committee of the University Medical Center (Web: https://umc.org.kz/en/?ethics-commission=post-2, accessed on 1 December 2025) approved the study (approval protocol #8/2024/ПЭ of 28 August 2024; monitoring and re-approval protocol #1/2025/ПЭ of 12 February 2025; Board Affiliation: University Medical Center). The committee confirmed that all methods were performed in accordance with the Declaration of Helsinki and the guidelines of the Council for International Organizations of Medical Sciences (CIOMS). Patients were not physically enrolled in the study.

For the definition of the term “Dysfunction of overweight”, lipids in the body perform various functions, such as an energy source, a shock-absorbing cushion for organs, an insulating and structural function, a fat depot, and the adsorption of various substances [33,52]. Overweight is a part of lipids that represent a depot in the form of fat reserves. Overweight serves as a source of energy in the absence of available food. Consequently, overweight dysfunction occurs when the body does not demand excess body weight, which leads to the interference of adipose tissue in the body’s metabolic processes [35,53]. Signs of dysfunction associated with excess weight may include inflammatory processes, hyperlipidemia/dyslipidemia, impaired oxidation–reduction reactions, increased temperature and/or blood pressure, and others [33,35,54]. Overweight is a dynamic parameter of the body, as it can be in a growth phase “growing Overweight” or in a stable state as “maximum or limit Overweight”, and these states must be distinguished.

**Trial Registration:** ClinicalTrials.gov NCT06410352 (5 August 2024): https://register.clinicaltrials.gov/prs/app/action/SelectProtocol?sid=S000EG8K&selectaction=Edit&uid=U0006MBT&ts=56&cx=-vph5l9 (accessed on 5 August 2024).

**Declarations:** The study was carried out in the Republic of Kazakhstan from 5 January 2025 to 28 February 2026.

## 3. Results

### 3.1. Search Results

The initial search included 3853 relevant articles and 46 pre-printed published articles. After duplicates were identified, 1761 records were removed. After the titles/abstracts/texts were evaluated, 1792 articles also were excluded. In total, 346 full-text articles were assessed for eligibility. From these, 204 articles were excluded for the following reasons: 52 articles did not measure adipocytes; 42 articles did not examine the relationship between excess BMI/obesity and adipocyte size; 29 articles did not provide sufficient data on glycemic parameters; and 81 articles contained non-quantitative parameters. The final sample comprised 142 quantitative articles (77 were observational studies and 65 were clinical studies). The literature screening is summarized in Figure 1.

### 3.2. Characteristics of Included Studies

We included both animal and human studies because the study inclusion terms underlie the development of all biological organisms (including humans). All the studies were published between January 2000 and January 2026 (26 years). In total, 113 human studies and 29 animal studies, including 56 systematic reviews/meta-analyses (a total of 142 articles), were included in the analysis. Table 1 summarizes the results of the relationship between overweight/obesity and/or increased adipocyte size (exposure) and increased HbA1c, insulin, and the development of IR and T2DM (outcomes) based on data from 77 original epidemiological/observational studies, including systematic reviews and meta-analyses.

Overweight and obesity are distinct pathophysiological and clinical conditions. But not every person that can gain weight would be considered obese [55]. For some people, being overweight can already affect their body as a condition of obesity. We focused on individual overweight for each patient [52,56]. We paid special attention to overweight, which can grow or stop at the individual maximum achieved, and distinguished between these overweight. This phenomenon is associated with the concept of the “obesity paradox”. Therefore, we collected clinical studies in which patients with T2DM were either overweight or obese.

**Table 1:** The relationship between overweight/obesity and/or increased adipocyte size (exposure) and increased HbA1c, insulin, and the development of IR and T2DM (outcomes) based on data from original epidemiological/observational studies, including systematic reviews and meta-analyses.

Authors	Protocol	Study Design	Overweight/Obesity, Increased Adipocytes	HbA1c, HI, IR, T2DM
Human Studies				
Dundar, 2022 [2]	860 subjects	Cross-sectional study	Overweight/obesity	Elevated HOMA-IR, developed T2DM
Sarkar, 2019 [57]	650 subjects	Cross-sectional study	Overweight/weight gain	Developed IR, β-cell deficiency, developed T2DM
Berglund, 2016 [58]	331 subjects	Cohort study	Overweight/obesity	Elevated HbA1c, developed HI
Cotillard, 2014 [36]	295 subjects	Cohort study	Overweight/increased adipocytes	Elevated HbA1c, developed HI, T2DM
Vertemati, 2008 [59]	56 subjects	Clinical controlled study	Overweight/increased adipocytes	Developed IR, T2DM
Fang, 2015 [28]	30 patients	Clinical controlled study	Overweight, increased adipocytes	Elevated HbA1c, HOMA-IR, developed T2DM
McLaughli, 2014 [60]	148 subjects	Clinical controlled study	Overweight/increased adipocytes	Elevated HOMA-IR
Pasarica, 2009 [34]	260 patients	Clinical controlled study	Overweight/increased adipocytes	Elevated HOMA-IR, developed T2DM
Ricci, 2015 [9]	22 studies, 4160 subjects	Meta-analysis	Overweight/obesity	Elevated HbA1c, HOMA-IR
Musilanga, 2024 [3]	30 studies	Systematic review, meta-analysis	Overweight/obesity	Elevated HbA1c, HOMA-IR, developed T2DM
Tahrani, 2022 [10]	55 studies	Systematic review	Overweight/obesity	Elevated HbA1c, HOMA-IR
Zhao, 2023 [15]	12 studies	Systematic review	Overweight/obesity/increased adipocytes	Elevated HbA1c, HOMA-IR, lipotoxicity
Villagrán-Silva, 2025 [27]	24 studies	Systematic review	Overweight/obesity	Elevated HbA1c, HOMA-IR, and miRNA
Ye, 2022 [61]	62 studies	Systematic review	Overweight/increased adipocytes/ectopic fat accumulation	Developed IR, T2DM
Papaetis, 2025 [29]	14 studies	Review	Overweight/increased adipocytes	Elevated HOMA-IR, lipotoxicity, developed T2DM
Nakamura, 2020 [62]	32 studies	Review	Overweight/increased adipocytes	Elevated HbA1c, HOMA-IR, lipotoxicity, developed T2DM, cardiomyopathy
Nakamura, 2024 [63]	47 studies	Review	Overweight/increased adipocytes	Elevated HbA1c, HOMA-IR, lipotoxicity, developed T2DM
Szablewski, 2024 [64]	14 studies	Review	Overweight/increased adipocytes	Elevated HOMA-IR, lipotoxicity, developed T2DM
Ferrannini, 2004 [65]	14 studies	Review	Overweight/obesity	Increased β-cell mass, elevated IR, developed T2DM
Szukiewicz, 2023 [66]	27 studies	Review	Overweight/obesity	Developed IR, T2DM, chronic diseases
Castillo, 2025 [67]	38 studies	Review	Overweight/intracellular lipid accumulation	Developed IR
Guria, 2023 [68]	53 studies	Review	Overweight/macrophage lipid infiltration	Developed IR, T2DM
van Vliet, 2020 [69]	24 subjects	Review	Overweight/increased adipocytes/ectopic fat accumulation	Developed IR, HI, developed T2DM
Lipke, 2022 [70]	35 studies	Review	Overweight/increased adipocytes	Elevated HbA1c, HOMA-IR, lipotoxicity
Mota, 2016 [71]	22 studies	Review	Overweight/increased adipocytes	Developed IR, lipotoxicity
Longo, 2019 [54]	19 studies	Review	Overweight/increased adipocytes	Lipotoxicity, developed T2DM
Ahmed, 2021 [72]	26 studies	Review	Overweight/increased adipocytes	Elevated HbA1c, HOMA-IR, lipotoxicity
Dahik, 2020 [73]	37 studies	Review	Overweight/increased adipocytes	Elevated HbA1c, HOMA-IR, lipotoxicity
Armato, 2025 [74]	1860 subjects	Review	Personal overweight	Elevated HbA1c, developed IR
Animal Studies				
Setayesh, 2019 [75]	(36 mice)	Animal controlled study	Overweight/obesity	Developed HI, DNA damage
Peyot, 2010 [76]	(Mice)	Animal controlled study	Obesity/lipid deposition	Developed IR, beta-cell failure
Bozec, 2016 [77]	(Mice)	Animal controlled study	Overweight/increased adipocytes	Developed IR, hypoxia, adipocyte apoptosis
Sakaguchi, 2017 [78]	(Mice)	Animal controlled study	Overweight/increased adipocytes	Developed IR, T2DM, metabolic syndrome
Ozcan, 2014 [79]	(90 mice)	Animal study	Overweight/obesity	Developed IR, T2DM
Verkest, 2011 [30]	(106 dogs)	Animal study	Overweight/obesity	HI, elevated HOMA-IR, changed adiponectin, leptin, beta-cell function

Abbreviations: HbA1c, glycated hemoglobin; DNA, deoxyribonucleic acid; HI, endogenous hyperinsulinism; HOMA-IR, the Homeostasis Model Assessment of insulin resistance index; IR, insulin resistance; T2DM, type 2 diabetes mellitus.

As body weight increases, the physiological strain on internal organs rises accordingly [25,75]. This weight gain is driven by the expansion of adipose tissue [15,29,53]. When adipocytes expand due to lipid accumulation, the increased cellular radius overburdens intracellular transport mechanisms. The distance for nutrient delivery from the membrane to the cell center increases, as does the distance for metabolic waste removal [63,80], eventually precipitating lipotoxicity and glucotoxicity [70,71].

This increase in cellular volume (cytomegaly) occurs through either functional hypertrophy or, as seen in T2DM, pathological fat accumulation, both of which disrupt the cell’s energy supply chain [36,81]. This cellular expansion (hypertrophy and hyperplasia) triggers intracellular mechanisms designed to limit further nutrient influx. One such mechanism may involve conformational changes in cellular receptors, reducing their sensitivity to the anabolic effects of insulin and leading to the clinical manifestation of IR [64,72].

**Figure 2:**
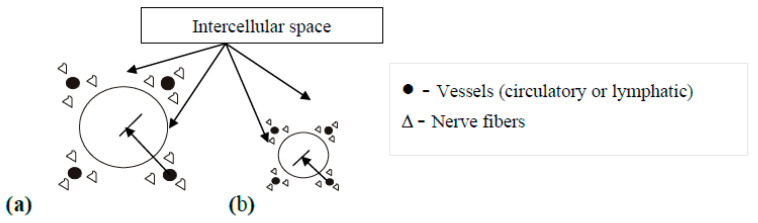
Schematic representation of a cell and the comparative relationship of its structural elements (vessels and nerves around the cell; cell radius, the distance from the center to the surface of the cell) with an increase in its size. Comparison of the structural elements of an enlarged cell (**a**) and a normal cell (**b**).

The forced infiltration of cells—including macrophages—with lipids [68] can eventually lead to apoptosis or necrosis [66,82]. Clinically, this may manifest as the progressive weight loss often observed in T2DM patients [54,83]. In the context of chronic disease, weight loss acts as a compensatory adaptive measure; similarly, the development of cellular IR serves as a necessary tool to prevent further nutrient overload [28,67,84]. Conversely, decompensated T2DM triggers pathological lipolysis—driven by “perceived” energy starvation and counter-regulatory hormones—leading to a search for alternative energy sources. Ultimately, IR at the receptor level represents a conformational shift that prevents further nutrient transport into an already overburdened cell [73,85]. The dynamic properties of these enlarged (cytomegaloylated) cells are compromised for the following reasons (Figure 2).

(1) ***Vascular Insufficiency:*** In tissues characterized by enlarged cells, the capillary density per unit of surface area becomes insufficient. The increased demand for blood supply and nutrients by these hypertrophied cells overburdens the transport capacity of the surrounding tissues and organs.

(2) ***Reduction in Intercellular Space:*** As cell size increases, the relative specific surface area decreases (surface-area-to-volume ratio), leading to a reduction in the available intercellular space. This contraction impairs intercellular metabolism and creates a relative deficiency in oxygen, regulatory mediators, and hormones.

(3) ***Organelle Scaling Imbalance:*** In hypertrophied cells, the stoichiometric relationship between intracellular structures is disrupted. The increase in total cell mass outpaces the biogenesis of mitochondria, endoplasmic reticulum, ribosomes, and other essential organelles. Consequently, the redox rate within the cytoplasm declines, leading to a deterioration of energy supply functions.

(4) ***Neurological and Trophic Compromise:*** The local nervous system and its conduction pathways become overburdened by the excessive regulatory demands of the enlarged tissue, resulting in the deterioration of trophic support for these cells.

Over time, these cytomegaloylated cells lose their functional adaptive value, transitioning from a compensatory state to a pathological burden on the organism.

**An enlarged cell is an unfavorable process for its normal functioning.** The consequences of this include the compression of blood vessels, impaired circulation, and impaired innervation. Increased cell size due to chronic nutrient consumption always occurs alongside the accumulation of overweight. Some studies have suggested that up to 25% of the population, even those who are not overweight, may have elevated HbA1c and blood insulin, leading to the further development of T2DM [74]. In the literature, this is called the “obesity paradox” [43,86], which we will discuss in more detail in the Section 4.

Table 2 presents the results of the impact of weight loss (intervention) on the regression of parameters (HbA1c, IR, T2DM are outcomes) based on 65 original randomized clinical and experimental trials, including systematic reviews and meta-analyses.

Weight-loss interventions resulted in significant reductions in all glycemic parameters (fasting glucose, HbA1c, blood insulin, IR), improvements in liver and kidney function, and normalization of lipids and blood pressure [25,45,48,87]. Regardless of the methods of intentional and targeted weight loss, one way or another, a decrease in the levels of HbA1c, HI, IR and T2DM is always observed.

**Table 2:** The relationship between weight loss (intervention) and parameters such as HbA1c, insulin resistance, and development of T2DM (outcomes) based on data from randomized clinical trials and animal studies, including systematic reviews and meta-analyses.

Authors	Protocol	Study Design	Weight Loss	Parameters of T2DM (HbA1c, IR)
Human Studies				
Franz, 2015 [5]	(6754 patients) Diet therapy	RCT	Weight loss ≥ 5%	Decreased HbA1c
Tahrani, 2022 [10]	(11 studies) Lifestyle	RCT	Weight loss ≥ 7.8%	Decreased HbA1c, HOMA-IR, T2DM remission
Tahrani, 2022 [10]	(12 studies) Pharmacotherapy	RCT	Weight loss ≥ 10%	Decreased HbA1c, HOMA-IR, T2DM remission
Lean, 2019 [20]	(149 patients) Lifestyle	RCT	Weight loss ≥ 10%	Decreased HbA1c, HOMA-IR, T2DM remission
Fonseca, 2019 [7]	(2432 patients). Semaglutide	RCT	Weight loss ≥ 7%	Decreased HbA1c, HOMA-IR
Zhao, 2023 [15]	(26 studies) Diet therapy vs. pharmacotherapy	RCT	Weight loss ≥ 5–10%	Decreased HbA1c, HOMA-IR
Buse, 2020 [45]	(American Diabetes Association и European Association for the Study of Diabetes) Pharmacotherapy	RCT	Weight loss ≥ 5%	Decreased HbA1c, HOMA-IR
Oshakbayev, 2017 [47]	(272 patients) VLCD	RCT	Weight loss ≥ 10%	Decreased HbA1c, HOMA-IR
Daniele [25]	(15 studies) Restriction diet	RCT	Weight loss ≥ 10%	Decreased HbA1c, HOMA-IR, SBP/DBP
Oshakbayev, 2019 [48]	(80 patients) VLCD	RCT	Weight loss ≥ 10%	Decreased HbA1c, HOMA-IR, NASH
Goni, 2017 [21]	(757 subjects) Low-fat diet vs. high-fat diet	RCT	Weight loss ≥ 5%	Decreased HOMA-IR
Banji, 2025 [88]	(56 studies) Pharmacotherapy	RCT	Weight loss ≥ 10–20%	Decreased HbA1c, HOMA-IR, T2DM
Albai, 2025 [87]	(256 patients) Pharmacotherapy	RCT	Weight loss ≥ 10%	Decreased HbA1c, HOMA-IR, T2DM, MASLD
Lingvay, 2020 [17]	(995 patients) Pharmacotherapy	RCT	Weight loss ≥ 5–10%	Decreased HbA1c, lipids, SBP/DBP, T2DM
Davies, 2022 [89]	(57 studies) Drug therapy, VLCD	RCT	Weight loss ≥ 5–20%	Decreased HbA1c, HOMA-IR, lipids, cardiorenal health, T2DM
Kashyap, 2022 [11]	(16 studies, 834 patients). VLCD	RCT and non-RCT, prospective controlled	Weight loss ≥ 5%	Decreased HbA1c
Horn, 2022 [12]	(45 studies) Lifestyle, pharmacotherapy, bariatric surgery	RCT and non-RCT, prospective controlled	Weight loss ≥ 5–15%	Decreased HbA1c, HOMA-IR
Ferrannini, 2004 [65]	(17 studies) Pharmacotherapy vs. bariatric surgery	RCT and non-RCT, prospective controlled	Weight loss ≥ 10%	Decreased HbA1c, HOMA-IR
Murphy, 2017 [90]	(33 studies) Bariatric vs. lifestyle	RCT and non-RCT, prospective controlled	Weight loss ≥ 7–10%	Decreased IR, adipocyte size, cardiometabolic diseases
Jooste, 2023 [91]	(11 studies and 1519 patients) Restriction diets	RCT and non-RCT, prospective controlled	Weight loss ≥ 5–10%	Decreased HbA1c, HOMA-IR, lipids, T2DM
Van den Burg, 2023 [92]	(9 studies) Different diets	RCT and non-RCT, prospective controlled	Weight loss ≥ 5–10%	Decreased HbA1c, BMR, T2DM
Schauer, 2012 [93]	(150 patients) Pharmacotherapy and bariatric surgery	RCT and non-RCT, prospective controlled	Weight loss ≥ 10–25%	Decreased HbA1c, HOMA-IR, lipids, SBP/DBP, T2DM
Ricci, 2015 [9]	(22 studies, 4160 patients) Bariatric surgery	Non-RCT prospective controlled	Weight loss ≥ 10%	Decreased HbA1c, HOMA-IR, T2DM remission
Tahrani, 2022 [10]	(12 studies). Bariatric surgery	Non-RCT prospective controlled	Weight loss ≥ 10–30%	Decreased HbA1c, HOMA-IR, T2DM remission
Cotillard, 2014 [36]	(74 patients) Bariatric surgery	Non-RCT, prospective controlled	Weight loss≥ 10%	Decreased HbA1c, HOMA-IR
Reinehr, 2004 [94]	(232 patients) Restriction diet	Non-RCT, prospective controlled	Weight loss ≥ 5%	Decreased HOMA-IR, cortisol
van Vliet, 2020 [69]	(24 studies) Restriction diet	Non-RCT, prospective controlled	Weight loss ≥ 20%	Decreased HOMA-IR, basal/ postprandial insulin secretion
Zhang, 2015 [95]	16 studies	RCT and non-RCT, prospective controlled	Weight loss/autophagy	Decreased HbA1c, HOMA-IR
Oshakbayev, 2026 [96]	(130 patients) Pharmacotherapy vs. bariatric vs. VLCD	Non-RCT, prospective controlled	Weight loss ≥ 10–20%	Decreased HbA1c, HOMA-IR cardiometabolic diseases, T2DM
Delrue, 2025 [97]	(25 studies) Restriction diet	Non-RCT, prospective controlled	Weight loss ≥ 10%	Decreased HbA1c, HOMA-IR, T2DM
Wei., 2025 [98]	(39 studies) Bariatric surgery	Non-RCT, prospective controlled	Weight loss ≥ 10–20%	Decreased HbA1c, HOMA-IR, T2DM
Animal Studies				
Di Daniele, 2021 [25]	(7 studies) Caloric restriction diet	Controlled studies	Weight loss ≥ 10%	Decreased HbA1c, HOMA-IR, SBP/DBP
Zhang, 2015 [95]	12 studies	Controlled studies	Weight loss/autophagy	Decreased HbA1c, HOMA-IR
Setayesh, 2019 [75]	(36 mice) Caloric restriction diet	Controlled studies	Weight loss	Decreased IR, inflammation, and DNA damage in internal organs

*Abbreviations*: HbA1c, glycated hemoglobin; DNA, deoxyribonucleic acid; HOMA-IR, the Homeostasis Model Assessment of insulin resistance index; IR, insulin resistance; NASH, non-alcoholic steatohepatitis; RCT, randomized clinical trial; SBP/DBP, systolic/diastolic blood pressure; T2DM, type 2 diabetes mellitus; VLCD, very-low-calorie diet.

## 4. Discussion

From a pathophysiological perspective, T2DM essentially manifests as elevated blood glucose because cells, already saturated with lipids (nutrients), are no longer capable of storing them [60]. Consequently, impaired glucose tolerance serves as an indirect indicator of cellular lipid overload, signaling that cells have reached their capacity for glucose uptake and subsequent conversion. It is important to note that cells do not store glucose directly; instead, excess circulating glucose is converted into fat for storage [99]. As these cells become increasingly saturated, they undergo a progressive increase in size [60,100].

Applying a fractal model to cellular geometry, the cell radius—defined as the distance from the membrane to the center—increases. This expansion significantly lengthens the transport pathways required for delivering nutrients and removing metabolic waste, further compromising cellular efficiency.

Chronic overeating coupled with overweight disrupt digestion and lead to metabolic intoxication and immune stress [101,102]. To clearly understand how cell enlargement leads to insulin resistance, it is necessary to briefly touch on the biochemistry of nutrients, as well as the physiology of digestion. Elevated blood glucose levels initiate glycogenesis and lipogenesis; once cellular glycogen stores reach their saturation limit, the pathway shifts toward de novo lipogenesis [99]. This physiological sequence is driven by the fact that glycogen is a more hydrophilic and bulkier macromolecule compared to lipids. In the context of a continuous nutrient surplus, lipogenesis is the more energetically efficient storage process. Lipids are less chemically reactive and more compact; for instance, the caloric density of one gram of fat is more than double that of one gram of glucose (or roughly equivalent to 3–4 g of glycogen when accounting for hydration) [99,100].

While glucose is highly reactive and occupies significant intracellular space due to its hydrophilic nature, lipids are anhydrous and structurally compact [103,104]. Furthermore, lipids represent the most structurally diverse class of nutrients, with an estimated 20,000 to 40,000 unique discrete structures identified in nature. Unlike proteins or carbohydrates, which are constructed from a limited set of standard monomers (such as 20 amino acids or a few simple sugars), lipids comprise a vast array of chemically distinct molecules.

Postprandial increases in circulating carbohydrates and lipids (cholesterol and triglycerides) drive the accumulation of newly synthesized fats within visceral and subcutaneous depots. When these primary storage sites become overloaded, excess lipids begin to accumulate as ectopic fat within functionally active cells (interstitial and intercellular spaces), triggering metabolic dysfunction. Significant focus is placed on postprandial glucose excursions, which play a critical role in sustaining chronic HI [105]. Furthermore, elevated HbA1c levels serve as a marker of the exhausted buffering capacity of erythrocytes [106]. The severity of T2DM clinical manifestations is directly proportional to the extent of involvement of these functionally active cells [79].

The body initiates several defensive responses to slow the lipid infiltration of these cells. These include hemodynamic and metabolic adjustments such as increased blood pressure, elevated body temperature, and heightened free-radical oxidation, all of which serve to accelerate the metabolic rate [61,107,108]. Additionally, the recruitment of the immune system leads to phagocytosis of excess lipids, resulting in the formation of foam cells. These cells subsequently adhere to vascular walls, contributing to the development of atherosclerotic plaques and the progression of IR [36,62].

The body’s natural process of “packaging” nutrients occurs in distinct, hierarchical stages. Evolution has favored mechanisms that allow the body to store energy economically, optimizing both spatial efficiency and metabolic conservation [109]. There is a finite capacity for nutrient accumulation in terms of both weight and volume. Once glycogen stores reach their physiological limit, the body shifts toward fat accumulation. While lipids are superior for long-term storage, glycogen serves as a critical “emergency” energy source, as it can be mobilized and converted into glucose much more rapidly than lipids [110].

The hydrophobic nature of a molecule dictates the efficiency of energy retention; hydrophobic molecules are more compact and chemically inert. Lipids, being highly hydrophobic and anhydrous, possess significantly higher energy density. For example, the β-oxidation of a single palmitic acid molecule yields 130 ATP molecules, whereas the complete oxidation of one glucose molecule yields only 36–38 ATP [111]. Atherosclerotic plaques are primarily composed of these highly hydrophobic lipids; their accumulation typically occurs in organisms that have lacked the physiological opportunity to mobilize and reduce excess body mass [112].

When target cells become overloaded with fat due to overweight, they use the resources of the body’s organs and tissues to support their own vital functions. For example, with obesity, the levels of many hormones (insulin, ghrelin, prolactin, cortisol, etc.) increase in proportion to body weight [94,113]. Excess adipose tissue imposes a heightened demand for vitamins, enzymes, hormones, and innervation to sustain basic cellular functions. Consequently, the internal organs must perform significant additional work—specifically in nutrient delivery and metabolic waste removal—to support these enlarged cells. While excess weight initially represents a growth phase, the body’s compensatory resources eventually become depleted. In the context of chronic obesity, the pancreatic islet system undergoes exhaustion; as β-cells fail to maintain the disproportionately high insulin secretion required by the excess body mass, the clinical symptoms of T2DM progressively worsen [61,114]. Overweight creates a state of relative insulin deficiency, where the pancreas cannot produce enough insulin to overcome the resistance of enlarged cells. Conversely, following weight loss, insulin sensitivity is restored [47,48,94]. This suggests that HI, in the context of excess weight, is a secondary compensatory response rather than a primary disorder.

The chronic underutilization of lipid reserves eventually leads to the development of atheromatosis and atherosclerosis Because physiological storage space is finite, the body undergoes a metabolic shift where unsaturated fats are transformed into saturated fats, and HDLs are converted into LDLs or VLDLs [53,115]. During the progression of weight gain, each subsequent stage is characterized by an increasing compaction of nutrients within the tissues [116]. Ultimately, the body reaches its individual “maximum overweight”—a metabolic ceiling or terminal overweight unique to each person [117].

Nature “has invented” a way to store energy as fat for use between meals or during natural disasters when food availability is reduced [4]. The main source of energy reserves are lipids (adipose tissue). It is no coincidence that the ratio of nutrients in the body—between fats, protein, and carbohydrates—is, on average, 2–8 (depending on the degree of obesity): 1: 0.25, respectively [118]. Fat accumulation is biologically and chemically preferable. The main factor controlling the rate of lipogenesis is the body’s nutritional status [119].

Chronic overeating combined with overweight expends/consumes the body’s potential energy on the processes of chemical digestion, absorption, transport, storage, and elimination of excess metabolites [101,102]. Increased insulin secretion by the pancreas is necessary to compensate for IR and maintain normal carbohydrate metabolism. In obese patients, the rate of insulin secretion is 3–4 times higher than in people with normal body weight, and HI is caused by both increased insulin secretion and decreased insulin clearance [69,76]. Gradually, the body’s compensatory resources deplete, and its additional synthetic and excretory functions reduce [57].

**Figure 3:**
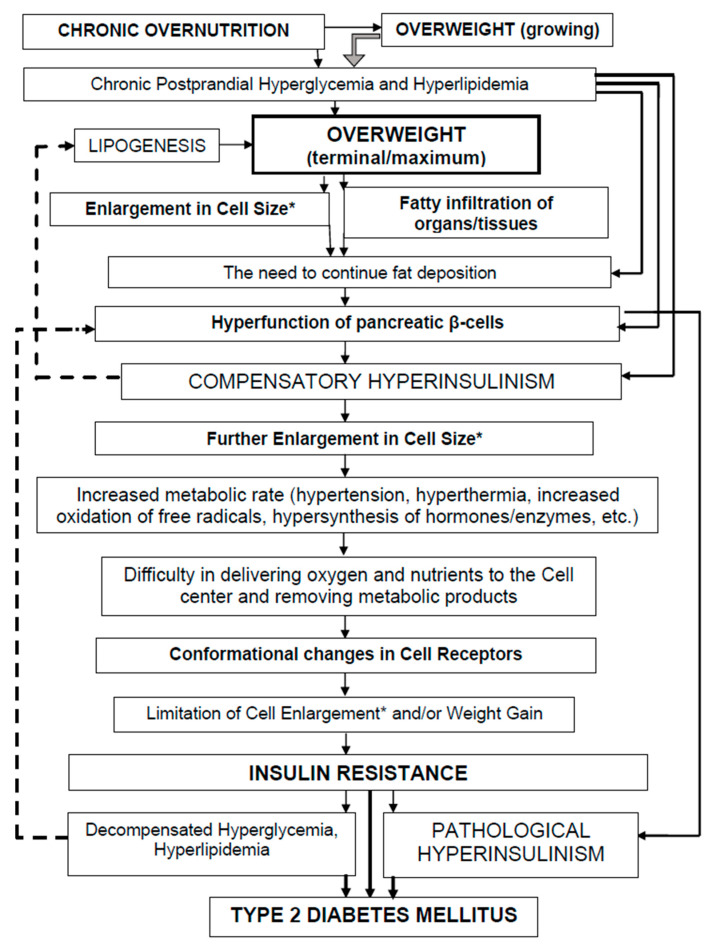
The concept of development of T2DM in the context of hyperinsulinism and insulin resistance against the background of overweight. * Adipocytes and other cells of the body with lipid deposition. Solid lines indicate a forward action; and dashed lines indicate a backward response.

HI is a physiological response to each food consumption [120]. Chronic overeating combined with persistent postprandial hyperglycemia and hyperlipidemia leads to overweight (Figure 3). The forced accumulation of fat in the body leads to an increase in cell size [59]. HI occurs as a compensation for the forced accumulation of fat by cells [69]. Pancreatic β-cells hyperfunction leads to compensatory HI. Elevated HbA1c is a sign of both HI and prolonged elevation of postprandial blood glucose levels [105,106]. When cells accumulate fat to their limit, a further increase can threaten their own destruction (death, apoptosis) [77]. This occurs in parallel when overweight is growing. HI induces conformational changes in cell membrane receptors, leading to IR to limit further nutrient accumulation [78]. IR refers to prolonged stress, i.e., prolonged release of insulin antagonists (catecholamines and cortisol) [113]. HI gradually loses its compensatory/adaptive value.

IR is the body’s pathophysiological response to the further entry of nutrients into the blood (hyperglycemia, hyperlipidemia) (Figure 3). IR is a rational response/process of the body that limits the further flow of nutrients into the cells. IR occurs when overweight reaches the terminal stage of growth. Stored fat reserves themselves require metabolic attention from the body, meaning they must be supported by blood circulation, thermoregulation, and anabolic/catabolic metabolism [78]. IR is a protective mechanism of cells against dangerous fat deposition and excess energy expenditure [77,121]. In turn, IR triggers a cascade of protective mechanisms that increase the rate of oxidation–reduction processes, raise the pulse and respiratory rate, etc. [78,90]. IR is an adaptive–compensatory mechanism that limits cell size [108]. In conditions of IR, the HI phenomenon suppresses lipolysis, which aggravates the progression of obesity and worsens IR itself [122]. Long-term HI, accompanied by overeating and overweight, depletes the secretory activity of pancreatic β-cells, which leads to the cellular glucose intolerance. The development of IR limits further accumulation of fat in cells [95,123]. A doom loop is created in which it is sometimes difficult to understand what is primary and what is secondary. Hyperglycemia, HI, and IR are different links in the same pathogenetic chain of development of T2DM.

A prerequisite for the development of IR is the dysfunction of overweight. Reaching one’s maximum (terminal) overweight depletes the reserve capacity of the body’s organs and tissues, including the pancreatic β-cells [65]. Therefore, IR is not a primary etiology but a secondary pathophysiological adaptation in T2DM. Clinical management should, therefore, focus not on the consequence (IR), but on the underlying cause—maximum overweight.

Figure 3 presents the concept of development of T2DM and its symptoms/syndromes. The figure shows that the development of T2DM is associated with dynamic changes in overweight; the development of HI and IR is associated with the dynamics of overweight growth, and the final stage of IR is associated with achieving overweight, which leads to the development of T2DM.

For the effect of overweight on the basal metabolic rate (BMR), overweight is the cause of the increase in overall energy costs [124]. Weight gain requires an increase in BMR as well as an increased food intake. Most of the BMR is spent on the consuming and processing food [125]. The body expends about 50 kcal/day of its own potential energy for every 100 kcal/day of additional food intake [126]. BMR accounts for 75–80% of total energy expenditure, and only 20–25% of energy expenditure is spent on external work such as physical and mental activity [127,128,129]. Daily excess food consumption increases the metabolic load on the body, as it increases both BMR and active metabolism [130]. Overweight speeds up BMR [131,132,133]. Overweight people are more likely to complain of fatigue [134]. One kilogram of excess weight deprives the body of approximately 50 kcal/day of daily energy expenditure [135]. On average, increasing food consumption by 175 and 204 kcal/day results in 100 kcal of energy expended per day [136].

The over-metabolism mode uses up the body’s excess “vital energy” [137,138]. Overweight is a useless cycle of consuming adenosine triphosphate [139]. The more active the metabolism, the higher the oxidative stress and the higher the oxidative function of the mitochondria [137,138]. When consuming excess protein, the body expends more energy, which increases the thermogenic effect to 25% of total energy expenditure [102]. A protein diet speeds up BMR. Overweight increases the total amount of metabolites [140,141]. Restricting food intake can reduce the BMR by up to 45% [142]. Weight loss reduces BMR and increases lifespan [127,141].

Consequently, there is a finite limit to cellular volume expansion. IR serves to protect the cell from nutrient oversaturation, restricting influx to prevent cellular structural failure or destruction. IR does not manifest instantly; rather, it is preceded and accompanied by hyperglycemia and HI, which represent progressive stages of metabolic dysfunction. In clinical practice, any sustained intentional weight reduction leads to a decrease in both IR and HI, often resulting in the remission of T2DM [87,88,96,97,98]. However, the speed and extent of this remission depend on factors such as patient age, gender, ethnicity, initial BMI, genetic predisposition, and disease duration [58,74].

Overweight correlates with the development of numerous chronic conditions, including T2DM, hypertension, allergic and inflammatory diseases, urolithiasis and cholelithiasis, non-alcoholic fatty liver diseases, liver fibrosis, and various malignancies [143,144,145]. Overweight is a constant and chronic consumer of insulin, limiting the reserve capacity of pancreatic β-cells. As an independent risk factor for T2DM, overweight must be the primary target of clinical intervention rather than the symptom (IR). Effective treatment of T2DM requires proactive weight loss; while weight gain negatively impacts energy balance and shortens life expectancy [146], weight reduction has been shown to increase longevity [147].

It is no coincidence that a new scientific field is developing with metabolic syndrome, which includes a cluster of pathological conditions such as abdominal obesity, impaired glucose tolerance, dyslipidemia/hyperlipidemia, hypertension, microproteinuria, and fatty liver diseases [29,39,78]. Although metabolic syndrome is currently rarely used by doctors in practice, it has the potential for further development, since its pathophysiological basis is in excess weight (abdominal obesity) [2,97].

Intentional weight loss reduces cardiovascular risks and the need for medications while improving glycemic metabolism [148,149,150]. Weight loss in patients with T2DM results in cost savings through reduced doctor visits, medication tests, sick days, emergency department visits, and hospitalizations, as well as reducing the risk of developing of chronic diseases, and has long-term economic benefits [91,151,152].

GLP-1 receptor agonists and GIP/GLP-1 agonists demonstrate significant weight loss, simultaneous improvements in blood pressure and blood sugar, and a reduction in cardiovascular events [89,153]. The more weight is lost, the better the fasting blood glucose, lipids, and blood pressure are [92,152,154,155,156,157,158]. After bariatric surgery, the need for antidiabetic and antihypertensive medications is often gradually reduced (under strict medical supervision) due to improved metabolic and cardiovascular health [98,159]. Significant weight loss allows for discontinuation of symptomatic medications due to the need to reduce the dose or completely discontinue previously taken medications [93,160].

Per the obesity paradox, some people with T2DM are not obese, and not all obese people develop diabetes. T2DM does not develop with a single fixed level of overweight; T2DM manifests with an individual level of overweight accumulation, namely its maximum value [161,162]. Thus, this is an indicator of the individual level of compensatory capabilities of each organism. One of the limits of the body’s compensatory function can be considered the moment of stabilization of maximum overweight, when body weight does not increase even with further overeating [74,130]. Only the volume of feces produced increases. As compensatory reserves are depleted, sooner or later a person with maximum overweight will develop T2DM [41,86].

An increase in adipocyte size, leading to IR and T2DM, is observed not only in obesity but also in overweight [64,74]. Each person has their own individual body weight and unique BMR, so the body’s potential energy is the potential ability to gain weight—the more potential energy a person has, the more weight they can gain [163,164], the more their body can increase in adipocyte size [64,70]. The body’s ability to gain weight is limited by its finite potential energy [165,166]. The limit of weight gain is the point at which the body weight cannot be increased further and the body weight stabilizes at its maximum point of overweight, which is called the “maximum overweight” or “maximum body weight”, and it is different for each person [140,163]. Body weight is an aggregated and integral indicator of the body’s energy reserves. Individual body weight and individual limits to weight gain explain the “obesity paradox”, which suggests that obesity in older patients with chronic diseases may be associated with reduced mortality among them [43,44].

A person capable of accumulating overweight is potentially strong, and the more excess body mass they can accumulate, the more individual potential energy they have. It is important to understand that this potential energy is expended on the biological/ biochemical/ biophysical maintenance of overweight. For instance, the more weight a weightlifter can lift, the more potential strength they have, but when they lifts the barbell to their maximum weight, all of their potential energy begins to be expended as kinetic energy in lifting and holding the barbell. The health status of people should not be compared based on overall/average body weight, because body weight is an individual variable for each person. Each person has their own individual maximum weight limit and unique potential energy reserves. It is necessary to compare the health level of the same person with different dynamics of their weight, which may indicate the degree of their individual potential energy. Weight loss creates “potential energy in the body”, which enhances physical and mental performance and promotes recovery from illness or weight regain. Individual limits to weight gain may explain the “obesity paradox” [43,44,74,86].

It is necessary to distinguish three modes of body weight change in adults.

1. In a state of excessive weight gain, the body begins to expend all potential energy on increasing/maintaining its weight (overweight growth mode). Weight can increase until the maximum excess weight limit is reached.

2. When the body is in the weight loss mode, there are two options: (1) intentional weight loss (due to restrictive diets, physical activity, bariatric surgery, medications, etc.); and (2) unintentional weight loss (due to chronic diseases, infections, stress and distress, other pathologies). Intentional (targeted) weight loss allows the body to increase potential energy, which promotes recovery and healing [129,141,167]. If there is an unintentional weight loss, then the body loses its own potential energy, which is an unfavorable indicator for health [43,86,162]. Almost any disease leads to unintentional weight loss because BMR increases [42]. Perhaps the body copes better with diseases if weight loss occurs. For instance, weight loss due to chronic and/or oncological diseases (unintentional weight loss) leads to the depletion of BMR, while intentional weight loss (restrictive dieting) helps maintain BMR [133,168,169].

3. Once the “maximum excess weight” is reached, the body gradually loses its own potential energy needed to maintain/provide for this excess weight (biological, biochemical, mechanical, etc.). The development of any pathology is a sign of a potential energy crisis. It is no coincidence that diseases are accompanied by unintentional weight loss [149,170]. Losing weight helps the body recover more quickly, while simply restricting food intake reduces BMR by up to 45% [142]. A restricted diet improves intestinal microbiota and vitamin synthesis in the intestine [171,172]. Intentional weight loss restores the body’s “potential energy” (which was expended for metabolic maintaining of overweight), which increases physical and mental activity and promotes healing from chronic diseases [46,47,149,150,173].

Almost any type of intentional weight loss improves anabolic processes, increases hemoglobin levels, improves metabolism in tissues and organs involved in hematopoiesis [17,45,89,91], increases HDL levels [92,154,155,156], and has an anti-osteoporotic effect [152,157,158]. We should use weight-loss methods that allow the body to conserve/save energy while simultaneously burning accumulated fat [151]. During weight loss, old fat deposits cause metabolic intoxication [53], which should be managed [46,47].

The evolutionary drive to accumulate fat mass was once a cornerstone of survival, as food scarcity was a constant threat throughout human history. This survival imperative—“eat whenever and wherever possible”—facilitated the buildup of vital fat reserves [145,146]. However, in an era of high food availability, this biological adaptation has contributed to a global obesity epidemic [131,144]. Society has yet to establish effective behavioral norms for managing dietary habits in an environment defined by a relative surplus of nutrients.

### Strengths and Limitations

The manuscript focuses on one biological aspect, such as the dynamics of overweight status, the state of adipocytes size can influence the development of T2DM, and its clinical and laboratory features in different ways, as well as discussing the vision of the origin of the “obesity paradox”.

This study has several limitations. The study was not designed as a meta-analysis. Published studies about the influence of dynamics of overweight status is very limited in scope and number. The study included systematic reviews, meta-analyses, and animal and human randomized studies. We used only reliable, scientifically validated sources (such as Web of Science, EBSCO, Scopus, Google Scholar, PubMed, Cochrane, и Ovid). We used the keywords “Overweight (growing)” and “Overweight (maximum)” though they were not available in MeSH on demand. There might also be publication/selection/analysis biases where valuable studies with negative results were not published or were published in journals without indexing in main bibliographic sources. A meta-analysis is required to more precisely quantify the correlation between adipocyte sizes and the pathogenesis of T2DM. Furthermore, meta-analyses are necessary to evaluate the longitudinal impact of various weight-loss modalities on IR and T2DM remission. Consequently, these results highlight a critical need for further empirical research to validate these pathophysiological mechanisms and refine clinical guidelines.

## 5. Conclusions

The results of our systematic review suggest three primary hypotheses concerning the pathogenesis and management of T2DM: (1) Cellular Protective Mechanism—excessive increases in cell size trigger conformational changes in cellular receptors, leading to IR. This is a protective response that prevents cellular structural failure and nutrient oversaturation. (2) Overweight as a Primary Driver—the accumulation of maximum overweight and increased cell size act as parallel processes that drive hyperglycemia and HI, eventually resulting in IR with T2DM. Overweight is confirmed as an independent risk factor for these metabolic disturbances and T2DM. (3) Reversibility through Weight Loss—intentional and targeted weight loss is shown to resolve symptoms of HI, IR, and T2DM, leading to clinical remission.

## Data Availability

The data are available from the authors upon reasonable request due to privacy. Those wishing to request the study data should contact the Principal Investigator of a research grant: Oshakbayev Kuat (Emails: okp.kuat@gmail.com; kuat.oshakbayev@umc.org.kz, phone +7-701-399-9394).

## Abbreviations

BMI	Body mass index
BMR	Basal metabolic rate
GLP-1RA	Glucagon-like peptide-1 receptor agonist
HbA1c	Glycated hemoglobin
HI	Endogenous hyperinsulinism
IR	Insulin resistance
RCT	Randomized clinical trial
SGLT-2i	Sodium–glucose transport protein 2 inhibitor
T2DM	Type 2 diabetes mellitus
